# Silicon / Perovskite Tandem Solar Cells with Reverse Bias Stability down to −40 V. Unveiling the Role of Electrical and Optical Design

**DOI:** 10.1002/advs.202401175

**Published:** 2024-06-18

**Authors:** Diego Di Girolamo, Olivier Dupré, Giuliana Giuliano, Jordi Veirman, Giuseppe Bengasi, Marina Foti, Cosimo Gerardi

**Affiliations:** ^1^ 3Sun S.R.L. Company of Enel Green Power Group Contrada Blocco Torrazze Catania 95121 Italy; ^2^ CEA, LITEN Department of Solar Technologies National Institute of Solar Energy Le Bourget du Lac F‐73375 France

**Keywords:** photovoltaics, reliability, reverse bias, silicon / perovskite tandem solar cells

## Abstract

The reverse bias stability is a key concern for the commercialization and reliability of halide perovskite photovoltaics. Here, the robustness of perovskite‐silicon tandem solar cells to reverse bias electrical degradation down to −40 V is investigated. The two‐terminal tandem configuration, with the perovskite coupled to silicon, can improve the solar cell resistance to severe negative voltages when the tandem device is properly designed. While perovskite cells typically exhibit early reverse bias breakdown voltages, the serial connection with silicon cells with large shunt resistances and high voltage breakdown limits their negative polarization and prevent the passage of large current densities when reverse biased. The importance of careful optical design is illustrated, with bottom‐limited conditions required to prevent the perovskite top cell from exploring its own breakdown. This aspect is of great importance in the case of partial shading events when the solar spectrum is richer in the IR components than the standard AM1.5G. Notably, 100% of efficiency retained after polarization at −40 V in different stressing conditions is observed. The results presented suggest that standard industrial bypass diode schemes may be compatible with silicon/perovskite tandem photovoltaics and provide new guidelines for the standardization of the stressing protocols.

## Graphical abstract

The electrical stability of silicon/perovskite tandem solar cells at −40V is investigated. The voltage breakdown and the shunt resistance of the silicon bottom cell should be maximised to protect the perovskite top cell. In the case of partial shadowing, the cell is stable for hours at −40V in bottom‐limited conditions while it shows a fast degradation in top‐limited conditions.

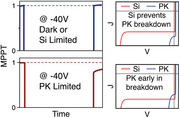

## 1 Introduction

A photovoltaic module consists of a series connection of solar cells. Within the string, a solar cell or a group of cells might experience reverse bias stress if shadowed during photovoltaic operations,^[^ ^1^ ^]^ acting as a power load,^[^ ^2^ ^]^ and potentially dissipating large amounts of energy. As a result, localized high‐temperature areas (known as “hot spots”) can arise,^[^ ^3^ ^]^ which may jeopardize the photovoltaic module integrity and reliability.^[^ ^4^, ^5^ ^]^

Silicon solar modules typically feature bypass diodes to reduce the amount of power dissipated by the shadowed solar cell(s).^[^ ^6^ ^]^ An alternative solution to reduce the dissipated power is to design the solar cells to have an intrinsically low breakdown voltage that does not result in device degradation.^[^ ^7^ ^]^ Conceptually, the latter approach is an analogue to having a bypass diode for each cell,^[^ ^8^ ^]^ which however would come with a cost penalty (every bypass diode adding ≈0.10–0.20 USD to the module price).^[^ ^8^ ^]^ Therefore, three bypass diodes are in practice introduced per module, one every 20–24 cells (depending on the module size). In such configurations, a single shadowed cell might be biased down to ≈−17 V (i.e., ≈ (24‐1) · Voc, with Voc (Open Circuit Voltage) ≈0.7 V) before the bypass diode kicks in, a value compatible with silicon technologies.

Thin film photovoltaic technologies are particularly sensitive to reverse bias stress,^[^ ^9^, ^10^ ^]^ since the standard bypass diode approach cannot be readily implemented in their typical module design^[^ ^11^ ^]^ with alternative approaches to mitigate the degradation from the reverse bias such as the optimization of the geometry of the cells or the layout of the modules still under investigation. This drawback (among others) possibly restricted the penetration of thin film technologies in the residential market, where optimal installation –, i.e., far from intermittent or dynamic sources of shadow – cannot be systematically guaranteed.

In addition to that, halide perovskite photovoltaics also suffer from the intrinsic instability^[^ ^12^ ^]^ of this class of materials due to electrical bias. This weakness arises from their ionic nature which allows ion migration, electrochemical reactions, and phase changes. To date, experimental results on reverse biasing single‐junction perovskite solar cells demonstrated that:^[^ ^13^, ^14^ ^]^

1. Perovskite solar cells have a relatively low breakdown voltage, in the range from −1 to −5 V (although a recent work from Ginger's group extended the V_bd_ down to −15 V^[^ ^15^ ^]^).
2. Perovskite solar cells are not stable after experiencing the breakdown, which commonly imparts a strongly shunted behavior (with the degradation only partially reversible).
3. The electrodes play a crucial role in defining the reverse bias stability of the solar cells, with metallic electrodes showing worse performances than Transparent Conductive Oxides (TCOs).

For the market entry of perovskite photovoltaics to be successful, it will therefore require a careful assessment of the stability of perovskite solar modules to reverse bias and their endurance to hot spots.^[^ ^16^, ^17^ ^]^

Recent work from Rand and De Wolf investigated this fundamental aspect and highlighted the interesting resistance of silicon/perovskite monolithic tandem solar cells to reverse bias stress.^[^ ^18^, ^19^ ^]^ This observation strengthens the hypothesis that silicon/perovskite monolithic tandem is the most interesting configuration for the market adoption of perovskite photovoltaics, also owing to the possibility to increase the efficiency of standard silicon modules beyond 30%. However, crucial insights are still required to properly design a perovskite silicon tandem solar module. In this contribution, we advance the knowledge on this critical topic by investigating the fundamental parameters of the tandem design which could affect the protective role from the silicon sub‐cell. In particular, we stress the relevance of the bottom cell electrical features, such as high shunt resistance (Rsh) and high breakdown voltage, in ensuring the protection of the perovskite top cell. Moreover, we show that in the case of partial shadowing, the current mismatch between the two sub‐cells strongly affects the protective role from silicon, and, therefore, a careful optical design would be required. We remark that the link between tandem stability and current mismatch is an aspect under serious scrutiny in various aspects,^[^ ^20^, ^21^, ^22^ ^]^ including the reverse bias, as hypothesized by Lan and Green.^[^ ^16^ ^]^ Our work provides the first experimental demonstration of the latter aspect, along with an investigation of the effect of the magnitude of the current mismatch at play in operative conditions. Notably, we show that we can reproducibly stress tandem solar cells down to −40 V for hours with negligible losses in their performances.

## 2 Results and Discussion

**Figure 1:**
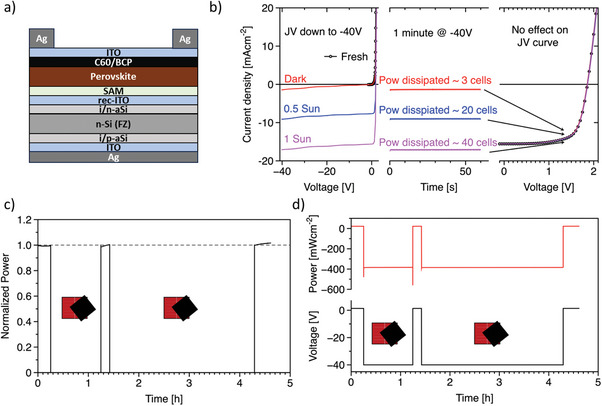
Stability at −40 V. a) Stack of functional films in the tandem solar cells investigated in this work. b) On the left, the current‐voltage (JV) curves in dark (red line), with 0.5 Suns at AM1.5G (blue line), with 1 Sun at AM1.5G (purple line) from 2.2 V to −40 V recorded with a step size of 0.5 V. On the same graph, we report with the black line with open points the dark JV curve recorded from 2.2 to −1.5 V with 0.05 V step size. In the center is shown the current recorded for 1 min while the cells are biased at −40 V under the same illumination conditions as for the JV curves on the left. On the right, the JV curves in the range 2.2–0 V measured after the stress test at −40 V. The JV curve of the fresh device is reported with the black line with open points. **c)** Normalized power transient of a tandem solar cell with fixed current set equal to the maximum power point current density (Jmpp). During the transient, we shadowed 50% of the area of the solar cell, inducing a fast and strong reverse bias down to −40 V (the instrumentation limit). After 1 h and then 3 h of the stress test at −40 V upon removing the shadow, the cell power is quickly recovered. **d)** Full‐scale power and voltage transients of the experiment in Figure 1c.

The solar cells investigated are based on an n‐type FZ wafer‐based HJT bottom cell and a pin perovskite top cell employing 2PACz as hole‐selective contact and C60/BCP as electron selective contact. The perovskite is a methylammonium‐free FA_0.79_Cs_0.15_Pb(I_0.81_Br_0.17_)_3_ composition. We report the architecture in **Figure** **1**a, and the details on fabrication are reported in the experimental part. In Figure 1b, we show the JV curves of a tandem solar cell from 2.2 to −40 V in the dark and at different illumination levels. In all cases, we did not observe an abrupt onset of the breakdown. Afterward, we kept the cell at −40 V for 1 min, obtaining a very stable current output, matching the −40 V data from the JV curves. Remarkably, the JV curves under AM1.5G measured right after the bias stress tests practically overlap with the initial JV curve, demonstrating that the PV parameters are left unaffected by the reverse bias step. Upon extending the stress test at −40 V, the devices still retain close to 100% of their initial efficiency (see Figure S1, Supporting Information, for 30 min and 16 h stress tests). It is interesting to note that the first JV curve immediately after biasing at −40 V features a small Voc loss, which is recovered in only a few fast JV scans. Interestingly, the Short‐Circuit current density (Jsc) is not affected (at least within the duration of this stress test). The conservation of the Jsc after a partial shading event would allow the solar cell to immediately resume producing power as soon as shadowing is removed. In case of a severe Jsc drop, the solar cell would remain pinned in reverse bias,^[^ ^23^ ^]^ increasing the risks of hot spots and degradation. To confirm this point, we simulated the real case scenario, by reproducing the dynamic effect of shadowing a cell in a module string. In the experiment whose results are shown in Figure 1c,d, we set a fixed current density (close to Jmpp) to the illuminated tandem cell, and we recorded the voltage and power transients over time. This experimental setting mimics the driving force bringing the shaded solar cell in reverse bias in real PV operations (see the experimental part for an explanation on this point). To induce partial shadowing, we covered 50% of the solar cell with a small piece of opaque material. When the cell is partially shadowed it immediately enters the negative voltage region. In the transient here reported when we shaded the solar cell, the voltage spontaneously fell at −40 V, which corresponds to the limit of our measurement instrument, and it is a realistic value as discussed later. Notably, upon removing the shading object after 1 h and then 3 h, the cell quickly recovers the initial voltage and power output, highlighting the capability to restart producing power even after a severe reverse bias event, with power dissipated in the range of 400 mW cm^−2^.

**Figure 2:**
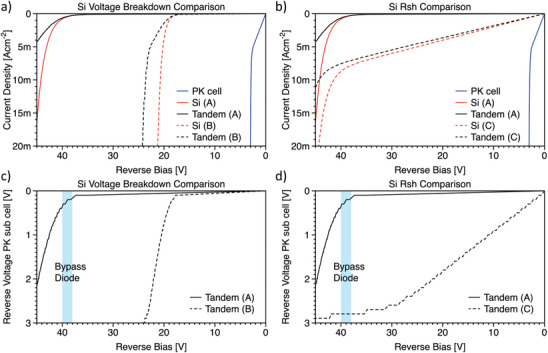
Effect of Electrical Features on Protective Role from Silicon a) Simulated JV curves in the negative voltage range for a perovskite solar cell with V_bd_ of ≈−3 V in blue, a silicon solar cell with V_bd_ of ≈−40 V in red, and a silicon solar cell with V_bd_ of ≈−20 V in red dashed line. In black, we report the JV curve resulting by connecting in series the perovskite cell with either of the two silicon cells. b) Simulated JV curves in the negative voltage range for tandem solar cell A (same as a) and tandem solar cell C, the latter composed with the same perovskite top cell and a silicon bottom cell with lower Rsh than case A. c) Voltage of the perovskite top cell as a function of the voltage of the tandem cells A and B (same as in a) in the range from 0 to −40 V. d) Voltage of the perovskite top cell as a function of the voltage of the tandem cells A and C (same as in b) in the range from 0 to −40 V. In (c) and (d) the light blue stripe indicates the voltage range where a bypass diode would kick in in a standard 60‐cell module with three bypass diodes.

By assuming the same architecture of standard silicon modules holds for tandem perovskite/silicon modules, we will have a bypass diode every 20 cells for a 60‐cell module. Therefore, the largest reverse bias that could be experienced by a shadowed cell will be ≈−38 V (assuming a Voc of 2 V for each cell). Therefore, a reverse bias experiment at −40 V as shown in this work could be a good figure of merit for the development of shadow‐resilient tandem solar modules. In this respect, it is important to understand in detail the protection of perovskite from the silicon sub‐cell. The series connection of the two sub‐cells is the physical origin of the robustness to reverse bias of perovskite/silicon tandem solar cells. In fact, the series connection imposes that the same current must flow across the two sub‐cells. Therefore, the perovskite cell will not experience its breakdown until the silicon sub‐cell reaches its own (i.e., the breakdown voltages (V_bd_) add, see **Figure** **2**a). This implies that the −40 V applied to the tandem solar cells in the experiments described in Figure 1, are not symmetrically distributed between the two sub‐cells. The first guideline is to implement a bottom cell with a large V_bd_ to ensure a tandem breakdown beyond −40 V. On top of that, it was shown in the literature that even the “early breakdown” can already damage perovskite solar cells.^[^ ^14^ ^]^ Therefore, a second guideline would be to develop a bottom cell with a large shunt resistance, with the goal of minimizing the current density at the voltage where the bypass diode starts to operate. To better illustrate those guidelines in Figure 2, we simulate the reverse bias behavior of three tandem solar cells employing the same perovskite top cell and three Si different bottom cells. We arbitrarily considered a perovskite top cell with a V_bd_ at ≈−3 V (in line with most literature^[^ ^12^, ^13^, ^14^ ^]^) and an Rsh of 500 Ω cm^2^. Concerning the bottom cells, we have:

- V_bd_ of −40 V and a Rsh of 500 kΩcm^2^
- V_bd_ of −40 V and a Rsh of 5000 Ωcm^2^
- V_bd_ of −20 V and a Rsh of 500 kΩcm^2^

The rationale behind those numbers is discussed in supporting information, Figure S2 (Supporting Information). In Figure 2a we report the simulated JV curve of tandem solar cells employing bottom cells A and B. In Figure 2b we show the JV plot of tandem solar cells employing bottom cells A and C, which differs in the Rsh.

To analyze the protection from the bottom cell, we simulated the voltage of each sub‐cell as a function of the reverse bias of the tandem solar cell. The respective roles of electric field and current density in determining the perovskite solar cell degradation in reverse bias have not been disentangled so far (to the best of our knowledge). Obviously, the two aspects are strongly linked, since a larger (negative) voltage implies a larger current density and electric field across the cell stack. However, when a solar cell enters its breakdown regime, tiny voltage increments result in a large amount of additional current flow. In this discussion, also following Rand and De Wolf,^[^ ^18^ ^]^ we focus on the voltage of the perovskite top cell, following the literature on single‐junction perovskite solar cells, where the reverse bias stability is typically analyzed as a function of reverse bias voltage.

In Figure 2c, we compare the voltage of the perovskite sub‐cell coupled to bottom cells A or B, which differ in V_bd_. The polarization of the perovskite cell is practically the same down to −20 V. Afterward, it increases more quickly for the perovskite top cell in device B, which will explore its breakdown well before the bypass diode protects it. On the other hand, in device A the perovskite top cell polarization is below −3 V, and the perovskite top cell does not go in breakdown.

In Figure 2d, we compare the voltage of the perovskite sub‐cell coupled to bottom cells A or C, which differ for the Rsh. In this case, in either device A or C, the perovskite top cell does not experience its own breakdown even when the tandem is at −40 V. However, in device C the polarization of perovskite is stronger than in device A, in the entire negative voltage range. At intermediate reverse bias polarization (e.g., when more than one cell is shadowed), device A would impart a more robust protection to the perovskite top cell, reducing the eventual impact of the perovskite early breakdown instability.

To summarize, the strongest protection arises when the Si sub‐cell has a very large shunt resistance and V_bd_, i.e., when it does not allow the current to flow when reverse‐biased. A large breakdown voltage can be achieved by tuning the wafer resistivity,^[^ ^24^ ^]^ with the larger breakdown voltage achievable with high resistivity wafers. The texture morphology^[^ ^25^ ^]^ as well might also impact the breakdown and early breakdown features. Therefore, the choice of wafers and the texturing recipes could need specific adjustments for the technological transition from silicon to silicon/perovskite tandem solar cells. On the other hand, along with depositing layers without pinholes, to achieve high shunt resistances in the bottom cell, it is fundamental to optimize all the steps of the fabrication workflow to avoid local defects, for instance avoiding as much as possible the marks due to the handling of the solar cells.

**Figure 3:**
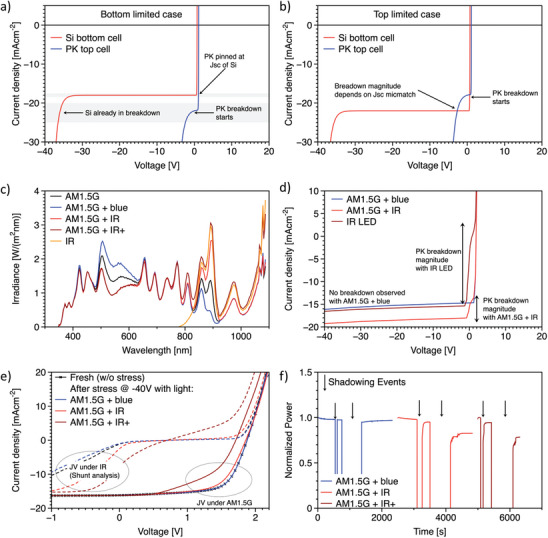
Effect of Current Mismatch on Protective Role from Silicon. Simulated JV curves, including the breakdown, for top (blue) and bottom (red) cells with 4 mA cm^−2^ mismatch for bottom a) and top b) limited cases. c) The spectra employed in the experiments reported in Figure 3. d) Experimental JV curves obtained with the spectra discussed in the main text and shown in Figure S1 (Supporting Information). When we use the spectrum “AM1.5G + blue”, we are in the case of (a), and the JV curve shows no evidence of breakdown down to −40 V. When we use the spectrum “AM1.5G + IR,” we are in the case of (b), and the JV curve shows the beginning of the breakdown of the perovskite top cell. In (d), we also report the case when we use only IR illumination, with the JV curve exploring a larger amount of breakdown of the perovskite top cell. e) JV curves at AM1.5G of tandem solar cells before (black) and after a stress test at −40 V for 7 min under different illumination conditions. Note that the black and blue curves overlap (the black curve falls exactly under the blue one). The dashed lines depict the JV curves under IR illumination only of the same tandem after the same stress tests. These measurements enable us to observe the variations of the shunt resistance and breakdown of the perovskite top cell (the slope around Voc is driven by the top cell's Rsh) f) Normalized power output transients recorded by imposing a fixed current close to Jmpp to a tandem cell under different illumination conditions. During the transients, half of the cell area is covered with a piece of paper to shadow from the solar simulator. Two different partial shading events were imposed, the first one ≈1 min long, and the second one lasting ≈10 min. This Figure illustrates the impact of the current mismatch on reverse bias resilience.

The previous analysis focused on a case where the cell was in the dark. Its conclusions remain valid when the cell is under illumination, but only if there is no mismatch in the photogenerated currents by each sub‐cell. In fact, the current mismatch between the two sub‐cells can strongly influence the protective role of the silicon bottom cell. An excessive current generation in the silicon cell (“top‐limited” condition) will result in a larger biasing of the perovskite top cell. This is sketched in **Figure** **3**a,b, where we show the simulated JV curves of two sets of sub‐cells in the case of the “bottom‐limited” condition (Figure 3a) and “top‐limited” condition (Figure 3b), by setting a large mismatch of 4 mA cm^−2^. To interpret these Figures, it is important to recall that the same current density flows across the two sub‐cells. In Figure 3a, the short circuit current of the silicon bottom cell is 18 mA cm^−2^. Therefore, when exploring the negative voltage region, the current will be pinned at this value until the silicon bottom cell undergoes its own breakdown. On the other hand, in Figure 3b, the larger current density available from the silicon bottom cell allows us to explore the breakdown of the perovskite top cell at intermediate negative voltages (or even at positive tandem voltages). To challenge this hypothesis,^[^ ^16^ ^]^ we conducted a reverse‐bias stress test on tandem solar cells illuminated with different spectra, shown in Figure 3c. In Figure 3d we show the JV curves obtained from the same solar cell after polarization down to −40 V under different illumination conditions:

1. AM1.5G + 2 mA cm^−2^ in the top cell −2 mA cm^−2^ in the bottom cell (AM1.5G + blue, inducing a “bottom cell‐limited” case)
2. AM1.5G −2 mA cm^−2^ in the top cell +2 mA cm^−2^ in the bottom cell (AM1.5G + IR, inducing a “top cell–limited” case)
3. IR LED, generating about the Jsc of the tandem but only in the bottom cell (inducing a strongly “top cell‐limited” case).

The JV curves in Figure 3d (and Figure S3, Supporting Information) closely follow the expectation from the modeling outlined in Figure 3a,b. In the case of a bottom‐limited cell, we observe a flat JV curve in the entire negative voltage range, following the JV curve of the silicon subcell. In the top‐limited case, the JV curve shows the onset of the breakdown of the perovskite top cell, until this negative current compensates for the mismatch. Then, going toward negative voltages the curve is flat as it is once again limited by the silicon subcell. The IR‐illuminated case is an extreme case of top‐limited condition since the perovskite top cell does not produce any current. In this condition, the perovskite cell explores its breakdown until it generates the same current available from the silicon cell (≈16mA cm^−2^ in Figure 3d). Therefore, depending on the amount of current mismatch, the degree of protection changes.

We performed a stress test to confirm that exploring the breakdown is harmful for the perovskite top cell, by imposing a fixed voltage of −40 V to the tandem cell for 7 min under the same illumination conditions explained above. In agreement with the results shown in Figure 1, the JV curve before and after the stress test in the “bottom cell‐limited” case perfectly overlaps (Figure 3e). When the stress test is performed in “top cell‐limited” conditions, the tandem device starts to suffer from a clear performance degradation (Figure 3d; Figure S4, Supporting Information). After 7 min of reverse‐bias stress test under “AM1.5G + IR” illumination, the tandem cell experiences FF and Voc losses, yet maintains above 80% of its initial efficiency in the first 60 s of tracking (the recovery is larger if we wait longer, see Figure S4, Supporting Information). However, after stressing the cell with only IR illumination, the performance loss is extremely severe, and less than 50% of efficiency is recovered after tracking. To confirm that it is the top cell undergoing degradation, we performed fast JV scans with only IR illumination right after measuring the JV curves under AM1.5G. When the tandem solar cell is illuminated with only IR light, the slope of the JV curve around Voc depends on the sub‐cell without photogeneration (i.e., the perovskite sub‐cell), thus reflecting its Rsh (see Figure S5, Supporting Information for a detailed discussion of this point). In Figure 3e, the *IR–JV* curves (dashed lines) evidence a sharp increase of the JV slopes around Voc after reverse bias in “top cell‐limiting” conditions, pointing to a strong decrease of the perovskite sub‐cell Rsh. This supports earlier reports of low robustness of PK cells to reverse biasing and strengthens the conclusion that the ability of the tandem device to sustain −40 V with no effect on the PV performances indeed stems from the protection from the silicon sub‐cell. To test the relevance of the current mismatch in realistic conditions when a solar cell is partially shaded (note that if a solar cell is fully shaded, there is no current mismatch since there is no photogeneration), we repeated the dynamic shading experiment discussed in Figure 1c, by covering half of the tandem cell area while illuminated with different spectral conditions and with fixed current density (Jmpp). In Figure 3f we show that the power output recovery after the shadowing event is immediate and complete in bottom limited conditions, while more and more performance losses occur when going toward top limited conditions (by increasing the IR component in the “AM1.5G + IR+” spectrum, Figure S1, Supporting Information), in agreement with the full area illuminated case. Notably, in the two top limited cases the power loss correlates with the time spent under partial shadowing. A slower recovery and lower plateau value in power production, in analogy to what is observed for the IR‐rich conditions in Figure 3f, was highlighted by Bowring et al^[^ ^13^ ^]^ upon cycling a reverse bias stress test. This fatigue effect only arises if the perovskite top cell starts degrading. In fact, we could not observe it either in Figure 1c,d or in a dedicated cycling test experiment presented in Figure S6 (Supporting Information).

It is important to note that here we highlighted a strong impact of the current mismatch, but light could interact with the electrical degradation in different ways. For instance, a dependence of the voltage breakdown on the illumination conditions was reported for CIGS technology, with blue light triggering the early breakdown.^[^ ^26^ ^]^ The set of data we collected so far does not suggest that a similar mechanism is in play here, however, this aspect deserves further investigation.

The above discussion indicates the current mismatch as a descriptive indicator of the perovskite top cell protection. By defining the current mismatch as $\Delta \;J_{\mathrm{SC}}=J_{\mathrm{SC}}^{\mathrm{PK}}\;-J_{\mathrm{SC}}^{\mathrm{Si}}$ we have the two cases:

(1) $$\begin{array}{c} \Delta J_{\mathrm{SC}}=\left\{\begin{array}{c} \geq 0;\;\mathrm{Si}\;\mathrm{limiting}\;\mathrm{case},\;\mathrm{the}\;\mathrm{perovskite}\;\mathrm{cell}\;\mathrm{is}\;\mathrm{protected}\;\\ \;\;\;\mathrm{depending}\;\mathrm{on}\;\mathrm{Rsh}\;\mathrm{and}\;\;\mathrm{VBD}\\ <0;\;\mathrm{PK}\;\;\mathrm{limiting}\;\mathrm{case},\;\mathrm{the}\;\mathrm{perovskite}\;\mathrm{cell}\;\mathrm{explores}\\ \;\;\;\mathrm{its}\;\mathrm{own}\;\mathrm{reverse}\;\mathrm{bias} \end{array}\right. \end{array}$$

**Figure 4:**
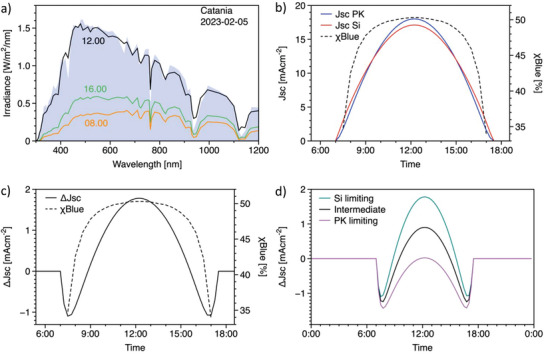
Spectral dependence of current mismatch a) Solar spectra at different times during a single day (the 5th of February 2023) in Catania. The reference spectrum AM1.5G is reported in full blue for comparison. All the spectra have been obtained by the pvlib simulation library (see experimental section). A comparison between the AM1.5G reference spectrum is provided in Figure S4 (Supporting Information) b) Jsc of perovskite (in blue) and silicon (in red) subcells across the day from which the spectra during a single day in Catania (the 5th of February 2023) have been calculated using the pvlib python library (see Experimental Section). The Jsc of the two sub‐cells have been integrated by employing the EQE data reported in Figure S4 (Supporting Information). The dashed line is the *x*_Blue_ parameter which accounts for the variation of the spectral density above and below 750 nm c) The same data from (b) are reported, however highlighting the difference in Jsc between the two sub‐cells, $\Delta \;J_{SC}=J_{SC}^{PK}\;-J_{SC}^{Si}$ d) The trend of Δ*J_SC_* for three different tandem solar cells with the EQE shown in Figure S5 (Supporting Information), with a different optical design at AM1.5G.

Notably, the current mismatch can vary substantially during the day, depending on the actual solar spectrum. Thus, this indicator allows us to investigate the level of protection from the reverse bias of the perovskite top cell when the tandem solar cell is in the field, exposed to spectra different from AM1.5G. To provide a general view on this aspect, we simulated the solar spectrum for Catania and Berlin on two different days: the 5 February 2023 and the 15 August 2023, to obtain insights at different latitudes and different seasons. The simulation is based on the Bird Simple Spectral Model,^[^ ^27^ ^]^ which neglects atmospheric events (rain, clouds), but still provides a realistic output in terms of the spectral distribution during the day. An example is given in **Figure** **4**a, where we compare the solar spectra in Catania during the winter at 8:00, 12:00, and 16:00. On the same graph, the AM1.5G spectrum is reported. Obviously, the irradiance in the morning and in the afternoon is much lower than at noon. However, the relevant aspect when dealing with the current mismatch is the spectral shape compared with AM1.5G. It is immediately evident from Figure 4a that the spectrum at 12:00 is very close to the standard one, while the two less intense are much richer in the IR region. To track this aspect, we introduced the parameter *x_Blue_* (the fraction of blue compared to AM1.5G), defined as follow:^[^ ^28^ ^]^

(2) $$\begin{array}{ccc} & & x_{\mathrm{Blue}}\\ & & =\frac{\int_{300}^{750}\mathrm{Irr}\;d\lambda /\int_{300}^{750}\mathrm{AM}1.5G\;d\lambda }{\left(\int_{300}^{750}\mathrm{Irr}\;d\lambda /\int_{300}^{750}\mathrm{AM}1.5G\;d\lambda \;\right)+\left(\int_{750}^{1200}\mathrm{Irr}\;d\lambda /\int_{750}^{1200}\mathrm{AM}1.5G\;d\lambda \;\right)}\\ & & \;\times \;100\;\left[\%\right] \end{array}$$

where “**Irr**” is the simulated solar spectrum and AM1.5G is the reference. When *x*_**Blue**_ is larger than 50% the solar spectrum is richer in the short wavelength region than the AM1.5G. In Figure 4b, we report *x*_**Blue**_ during the day along with the current density integrated over the different daily spectra for the two sub‐cells of a tandem device with the EQE in Figure S7 (Supporting Information). In the middle of the day, *x*_**Blue**_ is slightly larger than 50% (51%), while in the morning and in the afternoon, it deviates toward lower values accounting for the richer IR portion. Accordingly, the current density from the silicon subcell is larger than that from perovskite in the morning and in the afternoon (Δ*J*_SC_ < 0), while the opposite holds at noon (Δ*J*_SC_ ≥ 0). In term of reverse bias, this means that partial shading in the morning and in the afternoon would be more dangerous for the perovskite top cell. In Figure 4c, we report the trend of Δ*J*_SC_ during the day, showing that the current mismatch ranges from ≈−1 mA cm^−2^ (perovskite limiting) to ≈ +2 mA cm^−2^ (silicon limiting). This observation tells us that the perovskite top cell will explore its breakdown up to 1 mA cm^−2^ for less than 3 consecutive hours (both in the morning and in the afternoon) in the day under analysis. This picture strongly depends on the current mismatch at AM1.5G (i.e., the optical design of the tandem cell). In Figure 4d we compare three tandem solar cells with different optical designs, described in Figure S8 (Supporting Information). The tandem solar cell which is perovskite limiting at AM1.5G will produce a Δ*J*_SC_ < 0 for the entire day, so which implies more than 10 h with the perovskite top cell exploring its own breakdown (yet with variable magnitude) if it is shaded. On the other hand, the matched and silicon‐limiting solar cells will produce a Δ*J*_SC_ < 0 only for few hours in the morning and in the late afternoon. In supporting information (Figures S9 and S10, Supporting Information), we report the same analysis just discussed for a summer day in Catania and for a winter day in Berlin. In the summer in Catania (in low‐latitude locations) the solar spectra are always richer in the blue (*x*_**Blue**_ > 50%, Figure S9, Supporting Information), and therefore the three solar cells will experience negligible perovskite limiting conditions, Figure S7 (Supporting Information). On the other hand, the spectra in Berlin (in high‐latitude locations) are always richer in the infrared (*x*_**Blue**_ < 50%, Figure S8, Supporting Information), and the perovskite top cells will be less protected from silicon, Figure S10 (Supporting Information). However, also in this case the time spent producing a Δ*J*_SC_ < 0 can be limited to few hours if the tandem cell is designed as bottom limited at AM1.5G.

**Figure 5:**
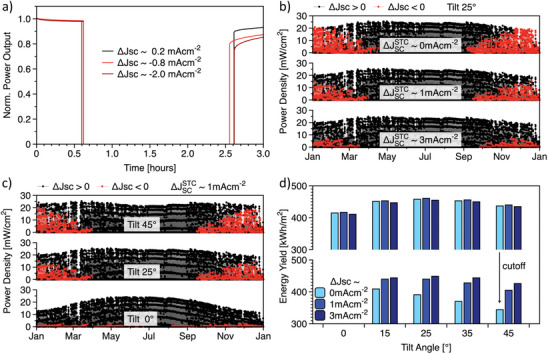
The tradeoff between current mismatch and energy yield. a) Partial shading stress test lasting ≈2 h with different mismatch conditions. It is shown that even a small amount of current mismatch induces a larger loss in terms of power output and a slower recovery kinetics. The current mismatch values and the duration of the stress test have been selected considering the variation of current mismatch across a day, as depicted in Figure 4c. b) Power output evolution during an entire year for the three tandem solar cells whose current mismatch is depicted in (a). Here, the points in black represent the power generated in conditions where the current mismatch is positive, Δ*J*_SC_ > 0. The red points represent the power generated in conditions where the current mismatch is negative, Δ*J*_SC_ < 0. In (a) and (b) the plane of the solar cell is tilted by 25°, south‐facing. c) Same graph as in Figure b, but considering the solar cell with ≈1 mA cm^−2^ of current mismatch in standard conditions and varying the tilt of the solar cell plane, always south facing. d) The simulated energy yield of the tandem solar cells with different current mismatches at standard conditions and different tilts. The upper row reports the total energy yield, while the lower row reports the energy yield obtained if the solar cell is disconnected when the current mismatch is negative, and eventual partial shading would become more dangerous.

The above analysis points toward a not negligible probability of spending at least two consecutive hours in non‐protected conditions (Δ*J*_SC_ < 0) in case of partial shading events. To gain a better insight into the stability risks of such circumstances, we repeated the stress test discussed in Figure 3f, extending the time scale and limiting the current mismatch within −2 mA cm^−2^. Notably, after ≈2 h of the stress test shown in **Figure** **5**a, we can observe a clear correlation between the magnitude of the current mismatch with the initial loss (i.e., immediately after the shadow is removed) and the recovery kinetics. This observation, linked to the spectral evolution outlined in Figure 4, calls for a more detailed investigation of the effect of the optical design under AM1.5G on the time spent in non‐protected conditions, an aspect that is not yet considered in the standardized stressing protocols (the IEC 61215‐2 norm or the ISOS protocols for perovskite^[^ ^29^ ^]^). We performed energy yield simulations and correlated the power production with the current mismatch at standard conditions, exploring the different degrees of bottom limited conditions, namely: current matched conditions (Δ*J*_SC_^STC^ = 0 mA/cm^2^), slightly mismatched conditions Δ*J*_SC_^STC^  ≈1 mA cm^−2^, equivalent to the experimental EQE in Figure S7 (Supporting Information) and strongly mismatched conditions (Δ*J*_SC_^STC^  ≈ 3 mA cm^−2^). For every case, we evaluated different tilt for south facing modules. The weather conditions are obtained from a typical meteorological year (tmy) obtained from the free online PVGIS software.^[^ ^30^ ^]^ The temperature of the solar cell is calculated with the Ross model.^[^ ^31^ ^]^ In Supporting Information (discussion and Figures S11–S16, Supporting Information) and in the Experimental Section, we provide a detailed description of the methods for the energy yield calculations and the possible impact on the results hereafter presented. In Figure 5b, we plot the power production (every point representing a single hour of production) for the three different current mismatch levels at standard conditions. In black, we reported the hours where the spectral distribution of the solar radiation would induce a Δ*J*_SC_ > 0, while red points indicate solar spectra conditions leading to Δ*J*_SC_ < 0. It is possible to observe a clear (and obvious) correlation between the density of red points with the optical design under AM1.5G. It is interesting to note that the red points are mainly localized in the winter months. This aspect can be explained by the variation of the average photon energy throughout the year, shown in Figures S11 and S12 (Supporting Information) with the 300–4000 nm integration limit. Interestingly, the power production profile across the entire year is only slightly affected by the current mismatch under AM1.5G. In fact, we obtain an energy yield of 458 kWh m^−2^ for Δ*J*_SC_^STC^ = 0 mA cm^−2^, 461 kWh m^−2^ for Δ*J*_SC_^STC^ = 1 mA cm^−2^ and 455 kWh m^−2^ for Δ*J*_SC_^STC^ = 3 mA cm^−2^. A weak dependance on the current mismatch in standard conditions of the energy yield has been recently discussed by Tomsic,^[^ ^32^ ^]^ who analyzed this aspect in several different locations around the globe. In our specific case, we can point to the compensating effect of the fill factor when mismatching the two sub‐cells in 2T tandem devices and the opposite impact of the temperature on the bandgap of silicon and of lead halide perovskite, which counteracts the current mismatch during the warmer (and sunnier) hours.^[^ ^33^ ^]^ In Figure 5c, we provide the same analysis for the slightly mismatched case (Δ*J*_SC_^STC^  ≈1 mA cm^−2^) evaluating the effect of the tilt angle. Notably, when the tilt is 0° (horizontal panel) the density of red points is greatly reduced. This is attributable to the larger impact of the diffuse radiation for horizontally placed solar cells, especially in the winter months. The opposite occurs when increasing the installation tilt to 25° (same condition of Figure 5b) and 45°. The energy yield decreases from 461 kWh m^−2^ at 25° tilt, to 440 kWh m^−2^ at 45° tilt and 417 kWh m^−2^ at 0° tilt. Therefore, the strategy of reducing the tilt to avoid Δ*J_SC_* < 0 conditions came with a sizeable penalty in terms of energy yield. The optimization of seasonal tilt strategies would not improve this situation, since the energy production with 0° tilt is strongly reduced in the winter months (see the power production profiles in Figure 5c), when the Δ*J*_SC_ < 0 conditions occur. Finally, we evaluated the possibility to preventively cutoff (shutdown) the power production when the solar spectrum would induce Δ*J*_SC_ < 0. Notably, this would have a very limited impact on the energy yield for solar cells with Δ*J*_SC_^STC^ = 3 mA cm^−2^, which would decrease from 455 to 449 kWh m^−2^, while it would strongly penalize cases with lower Δ*J*_SC_^STC^ and larger tilt angle, as shown in Figure 5d and in **Table** **1**.

**Table 1:** Energy Yield Simulation Results. In brackets the energy yield values with the shutdown approach.

Δ*J*_SC_^STC^ [mA cm^−2^]	EY 0° [kWh m^−2^]	EY 15° [kWh m^−2^]	EY 25° [kWh m^−2^]	EY 35° [kWh m^−2^]	EY 45° [kWh m^−2^]
0	415	451 (409)	458 (391)	453 (370)	437 (344)
1	417	453 (440)	461 (440)	456 (428)	440 (405)
3	411	447 (444)	455 (449)	450 (444)	435 (426)

## 3 Conclusion

In this work, we brought evidence that the silicon bottom cell can protect the perovskite top cell in 2T tandem solar devices. This result is encouraging for the reliability of perovskite‐based tandem photovoltaics, even maintaining the standard silicon module design, with three bypass diodes partitioning a 60‐series connected solar cells‐based module in 20 cell‐long substrings. Assuming a Voc of 2 V for tandem cells, the bypass diode will activate whenever the shadowed cell is biased beyond −38 V (Voc times 19 cells). Therefore, the stability at −40 V we report here has a relevant practical application.

However, we highlighted that the tandem solar cells' resistance to the reverse bias is not universal but depends on the electrical and optical design of the device. In fact, the protection from silicon is effective if the bottom cell features a breakdown voltage in the range of −40 V along with a high shunt resistance. Additionally, the tandem solar cell should be designed to prevent perovskite‐limiting conditions in terms of current mismatch. A large breakdown voltage can be achieved by tuning the wafer resistivity^[^ ^24^ ^]^ or tailoring the texture morphology,^[^ ^25^ ^]^ all aspects also impacting the presence of early breakdown features. To achieve high shunt resistances in the bottom cell it is fundamental to optimize all the steps of the fabrication workflow to avoid local defects. Finally, the optical design should ensure bottom‐limited conditions even when the tandem is exposed to spectra with higher IR content (e.g., air mass such as AM2, AM3 as well as over irradiance effects^[^ ^34^ ^]^). We remark that a small current mismatch is compensated by the increase in FF,^[^ ^35^ ^]^ a positive factor in the tradeoff between efficiency and protection of the perovskite in reverse bias events. This is reflected in the energy yield simulations for south‐facing solar cells in Catania, where it is possible to minimize the number of hours spent with solar spectra inducing a current limited by the perovskite subcell with a minimal compromise on the energy obtained. The geometry of the solar cell installation also impacts the current mismatch distribution, however in this case we could not find a good compromise, even considering seasonal tilt strategies. Additionally, our results call for the development of innovative photovoltaics operational procedures, for instance using sensors to detect and avoid the simultaneous occurrence of partial shading with perovskite‐limited conditions.

We believe that the results reported in this work could be of great relevance in the design of useful standardized stress tests for perovskite‐based tandem photovoltaics. The actual norm, the IEC 61215‐2, does not consider different spectral conditions for the hot‐spot endurance stress test and so would miss the impact of current mismatch on the reverse bias stability. Similarly, the consensus statement for stability assessment of perovskite PV based on ISOS procedures^[^ ^29^ ^]^ prescribes conducting negative voltage stress tests in dark conditions. Hopefully, future upgrades of these protocols could benefit from our investigation, implementing the spectral variation during the reverse bias stress tests as routine prescriptions.

Obviously, further development on the stability of halide perovskite solar cells is also required to ensure long‐term stability, especially considering the thermal stability associated with hot spots, not discussed in this contribution, and which might have a strong impact on the current mismatch, given the opposite temperature dependence of the silicon and perovskite bandgap.

## Conflict of Interest

3Sun s.r.l. is a company with interest in the production and commercialization of photovoltaic modules.

## Supporting information

Supporting Information

## Data Availability

The data that support the findings of this study are available from the corresponding author upon reasonable request.
