# Supplementary material for: Silicon / Perovskite Tandem Solar Cells with Reverse Bias Stability down to −40 V. Unveiling the Role of Electrical and Optical Design
Source: Adv Sci (Weinh). 2024 Jun 18;11(31):2401175. doi: 10.1002/advs.202401175 (PMC11336948; doi:10.1002/advs.202401175)
Supplement: Supplementary file 1 — Supporting Information [file ADVS-11-2401175-s001.pdf]

ADVANCED SCIENCE
Open Access

Supporting Information

for *Adv. Sci.*, DOI 10.1002/advs.202401175

Silicon / Perovskite Tandem Solar Cells with Reverse Bias Stability down to –40 V. Unveiling the Role of Electrical and Optical Design

Diego Di Girolamo, Olivier Dupré*, Giuliana Giuliano, Jordi Veirman, Giuseppe Bengasi, Marina Foti and Cosimo Gerardi*

Supporting Information

Silicon / Perovskite Tandem Solar Cells with Reverse Bias Stability down to -40V. Unveiling the Role of Electrical and Optical Design.

Diego Di Girolamo[1], Olivier Dupre[2], Giuliana Giuliano[1], Jordi Veirman[2], Giuseppe Bengasi[1], Marina Foti[1], Cosimo Gerardi[1]

[1] 3Sun S.R.L., Company of Enel Green Power Group, Contrada Blocco Torrazze snc, 95121, Catania, Italy

[2] CEA, LITEN, Department of Solar Technologies – National Institute of Solar Energy, F-73375, Le Bourget du Lac, France

Experimental

Materials

PbI_2 (99.99%, trace metal basis, L0279) and $PbBr_2$ (>98%, L0288) was purchased from Tokyo Chemical Industry (TCI) and FAI from Greatcells Solar Materials. CsI (99.999% trace metal basis), N,NDimethylformamide (anhydrous, 98.8%), and Dimethyl sulfoxide (anhydrous, ≥99.9%) were purchased from Sigma Aldrich. 2PACz powder (>98.0%) was purchased from TCI. Anhydrous ethanol was purchased from Carlo Erba.

Perovskite cells fabrication

The HJT bottom cell/ITO substrates were treated with UV-Ozone during 30 min. The hole selective layer was deposited by spin-coating from a 2PACz powder solubilized at 1 mM in anhydrous ethanol. The perovskite precursor was prepared in a nitrogen filled glovebox by mixing PbI2, FAI, PbBr2 and CsI in a DMF:DMSO (4:1 vol ratio) to obtain a 1.6 M solution with the following formula: $Cs_{0.15}FA_{0.79}PbI_{2.44}$-$Br_{0.45}$, hence 6% Pb excess. The solution was kept at 40°C under magnetic stirring overnight. After that, the solution of perovskite was filtered (PTFE, 0.45 µm) and spin-coated using a 3-step spin-coating protocol: 200 rpm for 5 s, 1000 rpm for 10 s and finally 6000 rpm for 20 s. During the final step, 150 µL of chlorobenzene was dropped on the substrate 5 s prior to the end of the protocol. The crystallization was completed by post annealing at 100 ◦C for 1 h in a nitrogen atmosphere. The electron selective layer was then deposited in two steps in a vacuum chamber by evaporation. A 10 nm layer of C60 was first deposited followed by a 5 nm layer of BCP. The top electrode consisted of 100nm of ITO deposited by sputtering and 200 nm of evaporated Silver.

Tandem solar cells fabrication

Silicon heterojunction solar cells were fabricated from 280 um thick float zone (FZ) cSi wafer with CMP mechanically polished (CMP) and cleaned surfaces (commercial wafers). After additional cleaning/HF last/drying in a GAMA wet bench, (i/p) and (i/n) hydrogenated amorphous silicon stacks were deposited respectively on the back and the front side using an HELiA plasma enhanced chemical vapor deposition (PECVD) from Meyer Burger. Transparent conductive oxides (TCO) were subsequently deposited in an HELiA physical vapor deposition (PVD) from Meyer Burger. A thin TCO (<20nm) was used for the front side as recombination layer, while the rear side received a standard 100nm ITO layer.

The perovskite top cells were fabricated as described above.

Tandem solar cells characterization

The current density–voltage J(V) curves of solar cells are measured on a Keithley 2402 measure unit. A Wavelabs SINUS300 class AAA solar simulator is used to generate different illumination spectra. In the case of tandem and SHJ devices, the temperature is regulated to 25°C by mechanical contact with a metallic chuck containing Peltier elements.

Dynamic Shading Stress Test

To simulate an actual event of shading and its impact on the polarization of a solar cell we fixed a current density close to current density at maximum power point (Jmpp) obtained under full illumination conditions. This condition is close to what the solar cell experiences in the solar module, where the strings not shaded produce their maximum power current density, which forces the shaded cell in reverse bias. The shaded cell will explore its negative voltage region until it is able to produce the Jmpp or the bypass diode kicks in. If the bypass diode kicks in, the shaded solar cell will not produce the Jmpp, but a lower value depending on its JV relationship and the amount of area shaded. In our experimental setup, the instrument has a voltage limit of -40V. When the solar cell reaches -40V, it will not produce exactly the Jmpp, as if the bypass diode jumped in.

Modeling of JV curves

Reverse bias behavior of JV curve was simulated with Matlab. The breakdown was modeled with a simple exponential equation with the parameters tuned to get the approximate breakdown voltages of -3V (for perovskite), -17V and -38V for the different silicon bottom cells. This method does not intend to simulate any specific breakdown mechanism or to provide specific physical insights into the breakdown mechanism. The aim is to empirically analyze the series connection of solar cells with different V_{bd}.

Simulation of Current Mismatch with Solar Illumination

The solar spectra at different locations and in different days have been extracted by using the pvlib python library, which implement the Bird Simple Spectral Model. This library allow to select a vast range of input parameters, for our simulation we used the following ones (for Catania):

latitude = 37.509 °

longitude = 15.087 °

tilt = 30°

azimuth = 180° (South facing)

pressure = 101325 mbar

water_vapor_content = 1.4164 cm

tau500 = 0.084

ozone = 0.3438 atm-cm

albedo = 0.1

The explanation of all the parameters is detailed at https://pvlib-python.readthedocs.io/en/stable/reference/index.html

Our choice was to remain general, and we kept most values as the default. Similarly, the detailed optical properties of the various layers constituting the solar cells have not been considered through full optical simulations. More accurate simulations can be obtained by investigating in more details all the parameters described.

A time step of 15 minutes has been selected to scan an entire day. The solar spectra impinging on the cell surface were used to calculate the integrated Jsc of the two sub cell by using the EQE in figure S4 (experimental) and SI5 (experimental and calculated to simulate different optical design).

The temperature has been neglected for sake of simplicity. Obviously, given the opposite dependance on the temperature of silicon and perovskite bandgaps, we expect this parameter to play a crucial role in the mechanism here described.

For the energy yield simulation, we employed the same python library and the PVGIS online free software to gather true data for weather and irradiance. A time step of 1 hour has been selected. The efficiency of the tandem solar cell at every time point has been calculated by summing the voltages of the two sub cells at every current density value and taking the maximum of the product (current * sum of voltages). This is equivalent to the method [1]

The values used for the solar cell simulation are reported here. Those have been taken from Jost et al.[2] as starting point and modified to get an efficiency and PV parameters close to the solar cells whose EQE is reported in figure S7.

j0(Pk) = 1E-15 A/cm^2
j0(Si) = 1.8E-12 A/cm^2
n(Pk) = 1.44
n(Si) = 1.18
rs(Pk) = 5.0 Ωcm^2

rs(Si) = 3.0 Ωcm^2
rsh(Pk) = 1000.0 Ωcm^2
rsh(Si) = 5000.0 Ωcm^2

The efficiency of the tandem solar cells. It is possible to see the effect of the current mismatch on the FF and the Jsc.

ΔJ_{SC}^{STC} [mA/cm^2]	PCE [%]	Jsc [mA/cm^2]	Voc [V]	FF [%]
**0**	25.9	18.8	1.83	75.5
**1**	26.0	18.5	1.83	77.0
**3**	25.4	17.6	1.83	79.2

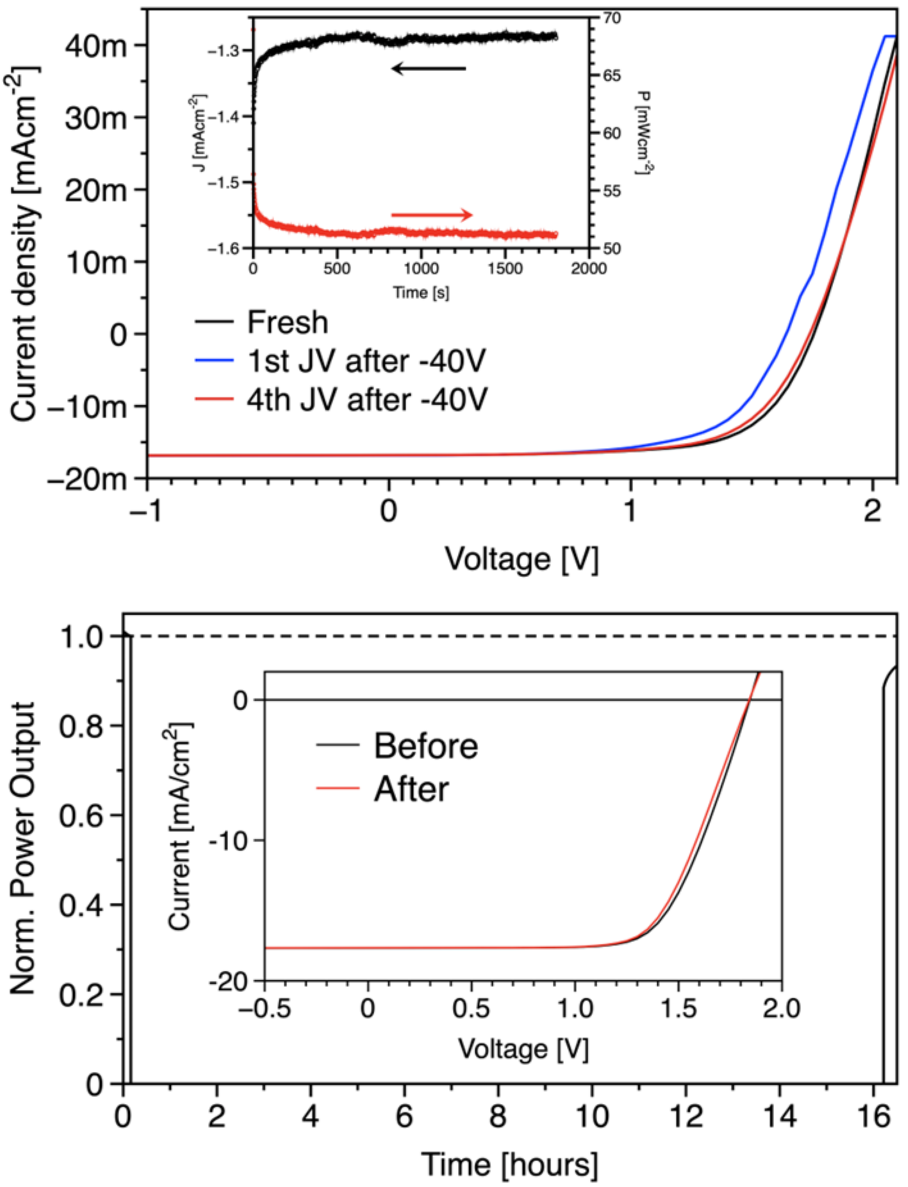

Figure S1 top JV curve of a tandem solar cell before (black line) and after 30 minutes at -40V in the dark. Several JV curves have been successively recorded after reverse biasing and in the legend, we highlighted the first one in blue and the last one (4th) in red, to show the fast recovery of the Voc and FF. In the inset, the current density, and the power density during the stress test. Note that the cell is placed on a chuck kept at 25°C through all the experiments. **bottom** 16 hours long stress test, showing a slow power output recovery. The JV curves before and after the stress test (which is from 15 to 3 times longer than the IEC norm) show a small loss in FF, due to increased Rs, yet maintaining the 94% in tracking (with the plateau not yet reached) and more than 98% comparing the JV curves.

The Voc dynamics is likely attributed to the contribution to the Voc of the perovskite top cell, since this PV technology is well known to show hysteresis and dynamic behaviors due to its ionic transport properties and peculiar defect chemistry. When a voltage is applied to a perovskite solar cell, its ionic/defect distribution is modified. During the reverse bias stress test, even if the silicon sub cells protect from the breakdown, the

perovskite top cell will be polarized at negative voltages. Negative polarization can impact the built-in potential due to ionic accumulation at the perovskite interfaces or can affect the defect density, as discussed by in ref 12 of the main text.

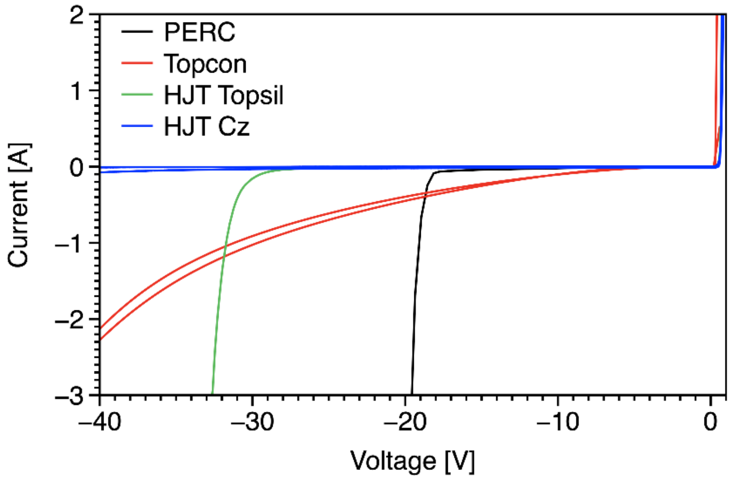

Figure S2.

Top) It is possible to observe that p-type PERC solar cells have a breakdown voltage compatible with tandem B in figure 2. While n-type Topcon and HJT show larger breakdown voltages.

Concerning the Rsh, 500Ωcm^2 is in within the range we usually get for the top cell, while Rsh in the range 5-500 kΩcm^2 are in the range for silicon bottom cells. As a comparison, the paper from Jost et al.[2] adopt similar values.

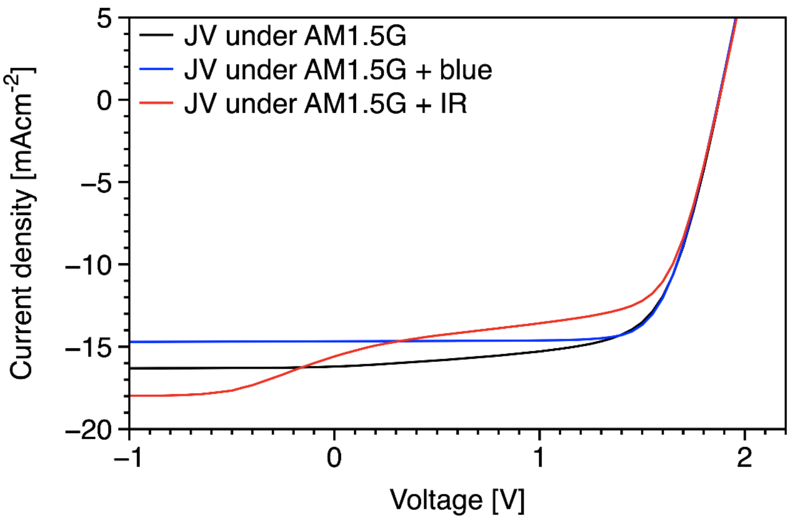

Figure S3. JV curves of tandem solar cells recorded with the same spectra employed for the stress tests discussed in figure 3e and 3f.

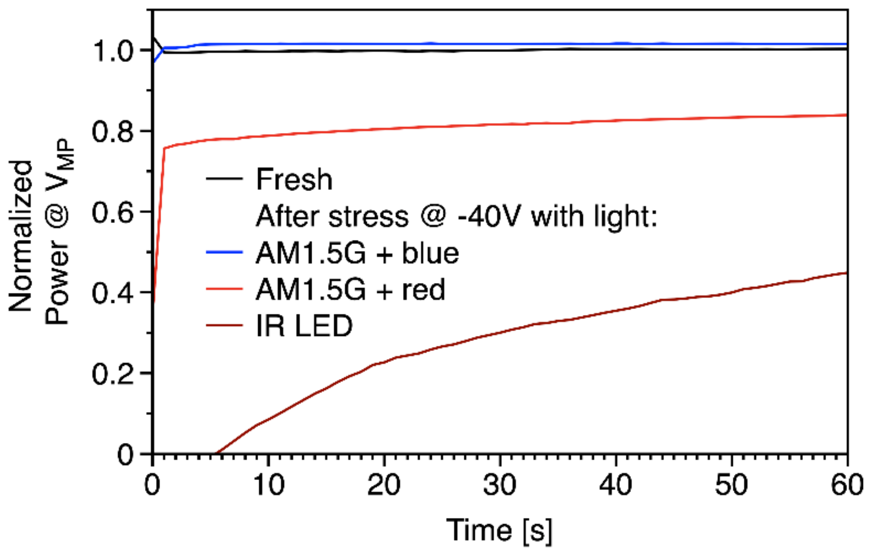

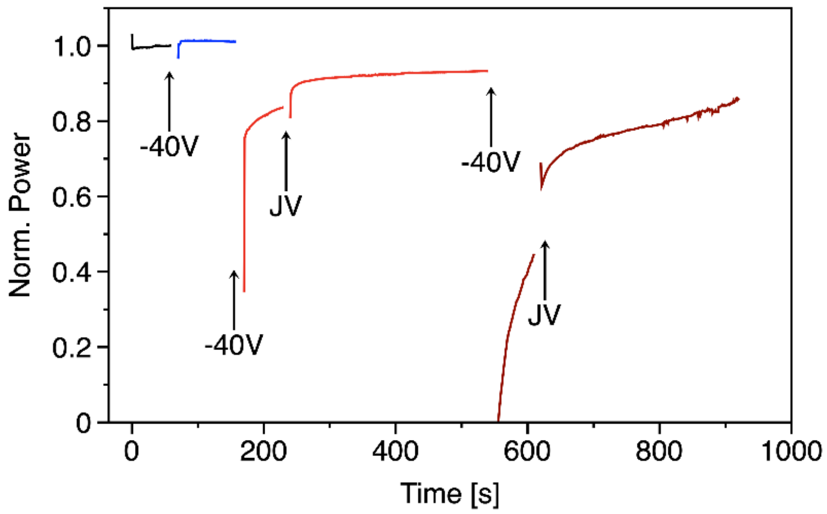

Figure S4 top) Normalized power transients at fixed voltage (the Vmpp of the fresh device) at AM1.5G for the same devices of figure 2c. Note that the power transient recorded after reverse biasing the cell under only IR illumination starts from negative values. The reason is that the transient is recorded at fixed voltage equal to the maximum power voltage of the fresh cell. Immediately after the reverse biasing, the Voc of the stressed cell is below this value. **bottom)** The complete power transients from the reverse bias stress test experiment discussed in figure 2c and S2 top. The color code follows the one from the main text. With the arrows we indicate that the transient was interrupted to conduct either a stress test (with "-40V" tag) or a characterization routine (with "JV" tag). The interruption time length are not to scale, we set 10 seconds to improve the readability of the figure.

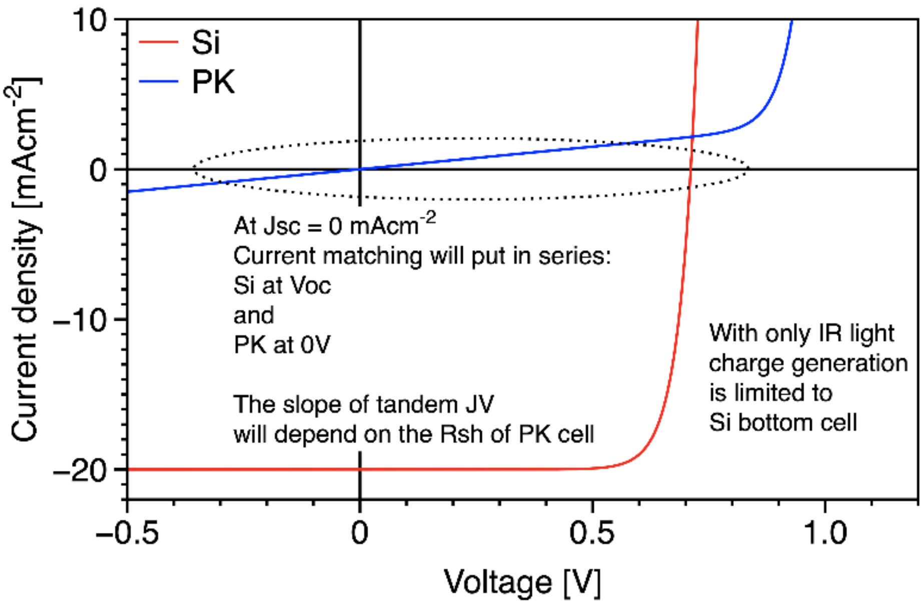

Figure S5 The case with only IR illumination is conceptually similar to the top limited case discussed in figure S2. However, this is a special situation because the perovskite top cell is in dark conditions. For small values of current density, the silicon cell will move along its JV curve around the Voc (i.e., small variation in voltage for large variation of current) while the perovskite top cell will move along its JV curve across its own short circuit (in dark) condition. Therefore, for such low current density values the JV curve of the tandem solar cells will be a good approximation of the perovskite top cell in dark, with slope proportional to the inverse of the shunt resistance.

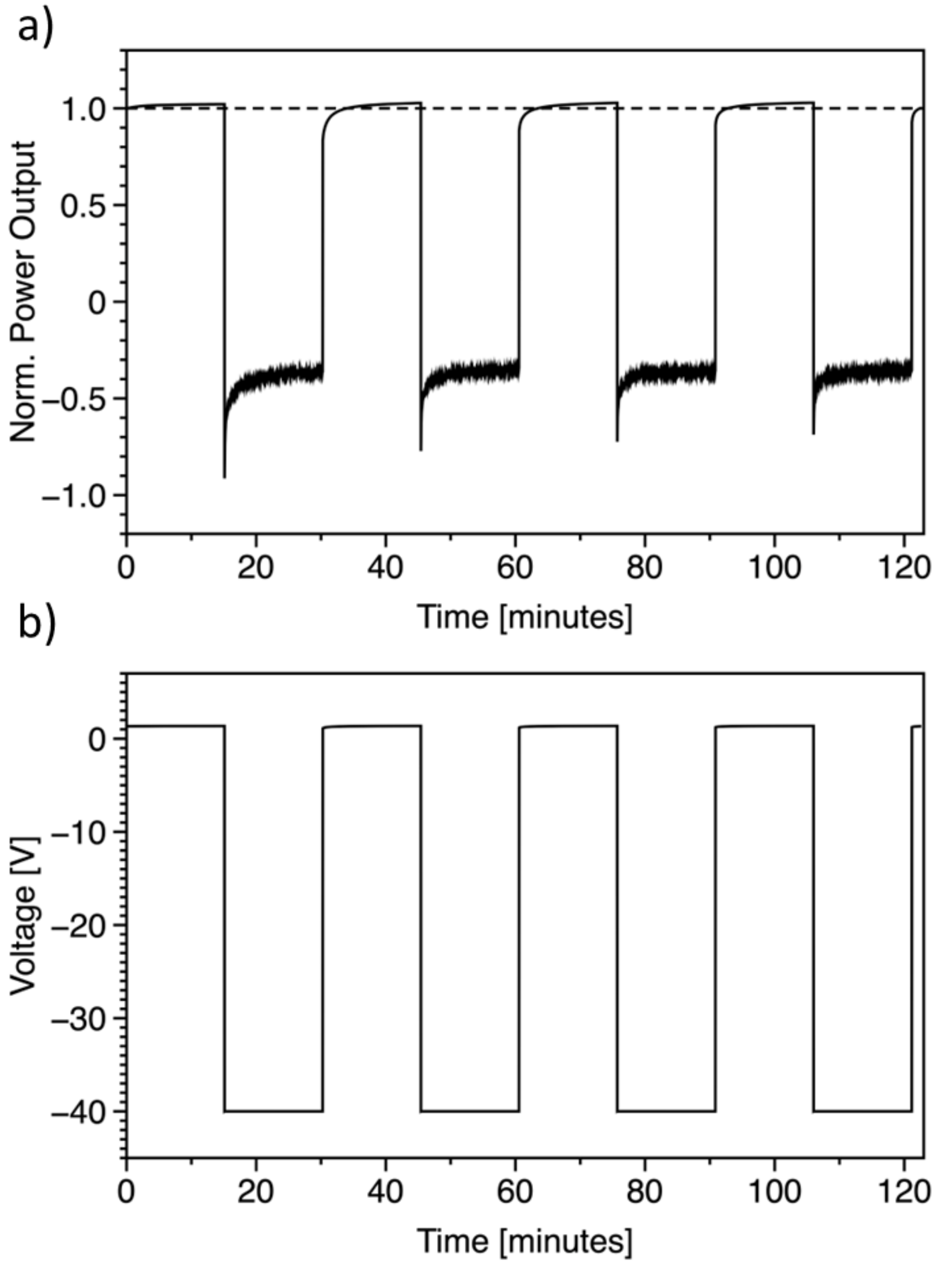

Figure S6 Cycling of the reverse bias stress test. In this experiment we explored if an additional degradation mechanism could arise from the cycling of the stress test. In fact, fatigue behavior has been evidenced in Bowring at al[3] , and we observed similarly in figure 3f. Here, several 15 minutes long stress tests have been performed, by maintaining a fixed current close to Jmpp and switching on and off the light of the solar simulator. The stress test drives the cell down to -40V in dark. In this condition, the perovskite top cell is protected, and within our observation time scale, we could not see the onset of any fatigue behavior. It is important to note that in figure 3f, a total of 8 minutes of stress test (divided in two different stress test for each of the AM1.5G+IR and AM1.5G+IR+ spectra) is enough to induce a sizeable loss in power and the fatigue effect attributable to the perovskite degradation.

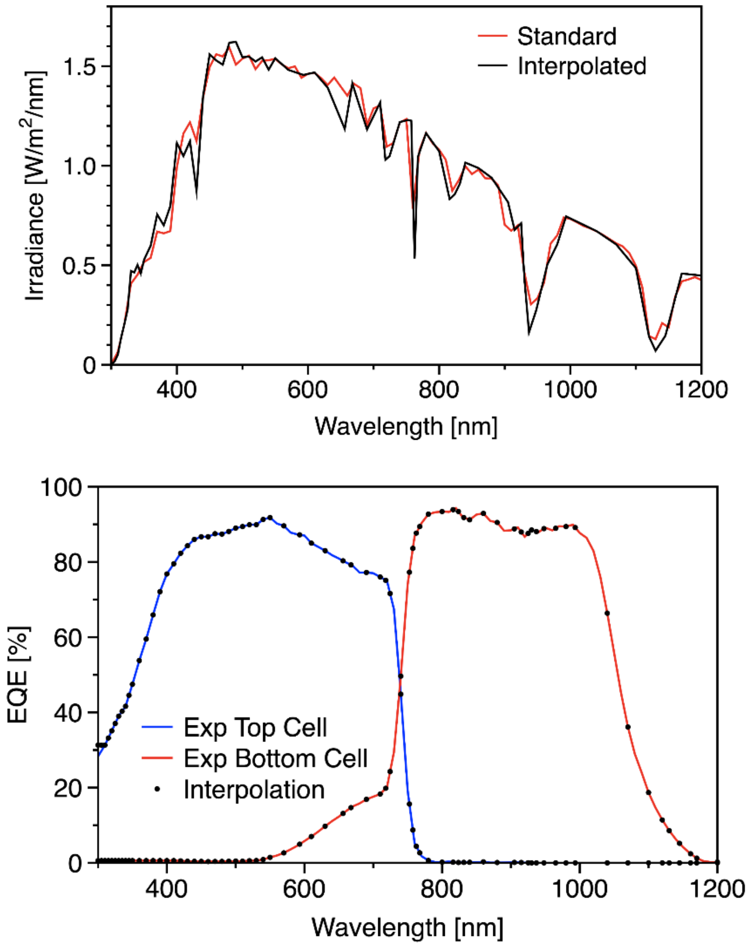

Figure S7 Top) The reference AM1.5g spectra and the one obtained from the pvlib python library used for the simulations in this work. **Bottom)** The experimental EQE as continuous line and the interpolation done for the data analysis with the pvlib library. There is an excellent agreement and the different wavelengths sampling has a minimal effect on the integrated Jsc (for both sub cells the integrated Jsc is about 0.2mAcm^{-2} lower, not affecting their relative magnitude).

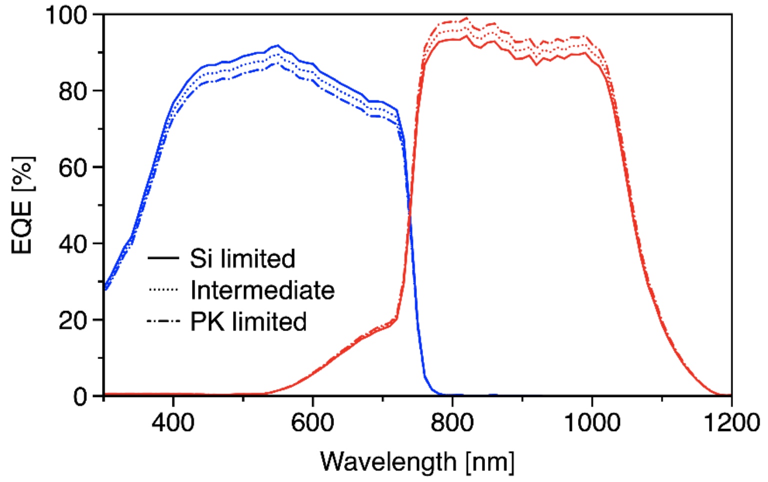

Figure S8 The EQE employed for the three cases discussed in the main text. The Si limited case uses the experimental EQE, which is scaled by 2.5% and 5% for the intermediate and PK limiting case. This approach has been selected to maintain a general view on the discussion in the main text. A detailed analysis considering the effect on the EQE and on the current mismatch of different configurations, materials, thicknesses and so goes on could be an interesting follow up of this general work.

The integrated Jsc values of the two sub cells are:

J_{SC}^{Si} = 18.2 mA/cm^2

J_{SC}^{PK} = 19.3 mA/cm^2

This tandem solar cell with 9cm^2 area performed as follows:

Voc = 1.830 V

Jsc = 18.7 mA/cm^2

FF = 74.9%

PCE = 25.6%

The efficiency of single junction perovskite solar cells and silicon solar cells with fabrication protocols similar to those employed in the tandem solar cells are as follows:

	Voc / V	Jsc / mAcm^{-2}	FF / %	PCE / %
PK SJ	1.160	20.1	73.7	17.2
HJT SJ - Cz	0.737	38.1	80.7	22.7

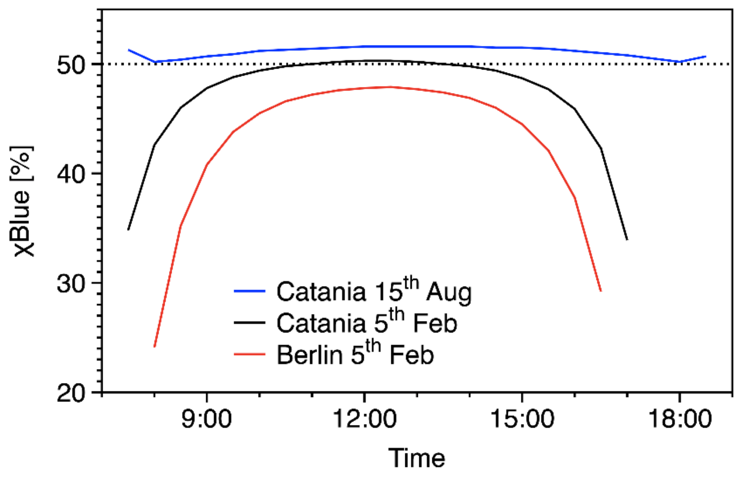

Figure S9 Analysis of the xBlue parameter in different locations and different days of the year.

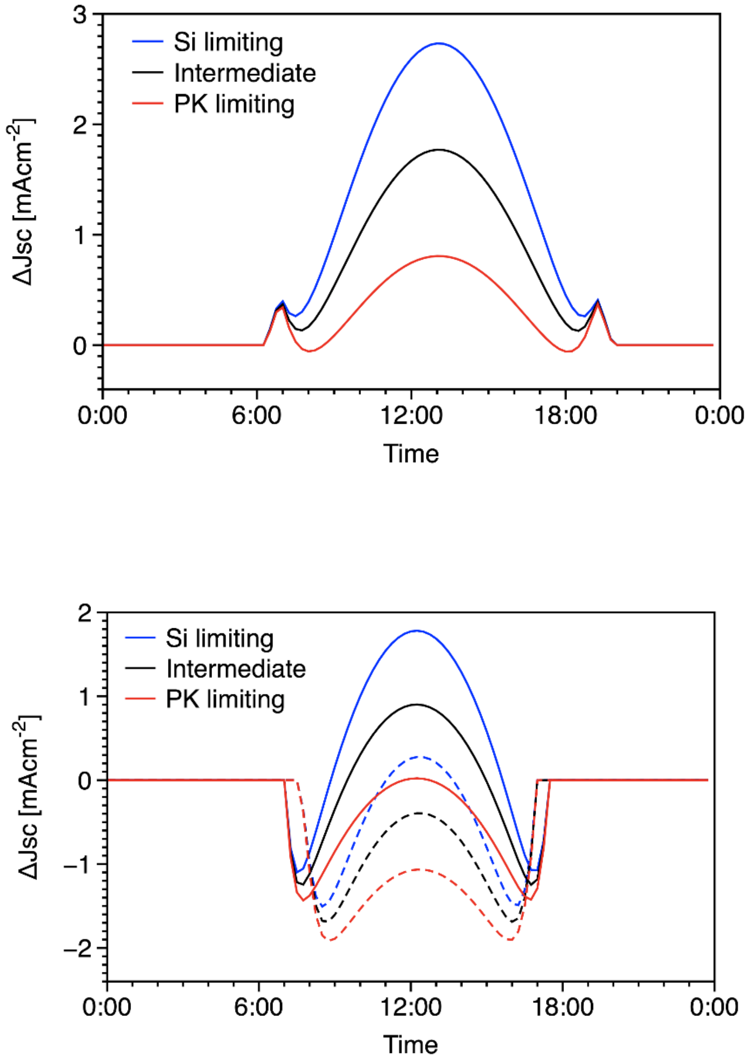

Figure S10 Current mismatch analysis for the 3 different cases of tandem solar cells obtained with the EQEs shown in figure S5. **Top)** The case of Catania on 15th of August 2023. In this day, the solar spectrum is always richer in blue (see S6) than AM1.5g, bringing the tandem cell always in bottom cell limited condition, thus in the most protective condition. **Bottom)** Comparison between Catania (continuous line) and Berlin (dashed line) on 5th of February 2023. The spectra in Berlin are richer in red than in Catania (see S6) and this put the tandem cells in perovskite limited conditions for more hours during the day. According to the effect of the current mismatch on the protection from silicon, the solar cells will be less protected from partial shadowing in Berlin.

Analysis of Energy Yield Model and Assumptions

Solar Spectra Evaluation across typical meteorological year.

The light impinging on the solar module is composed of direct and diffuse light. The direct light has a lower average photon energy than the diffuse light. Here, we report the average photon energy for the global, the diffuse and the direct radiation for different installation tilt angles. It can be observed that the global radiation attains APE values intermediate between direct and diffuse. The APE value is closer to the former, being the direct radiation more intense in standard clear sky conditions.

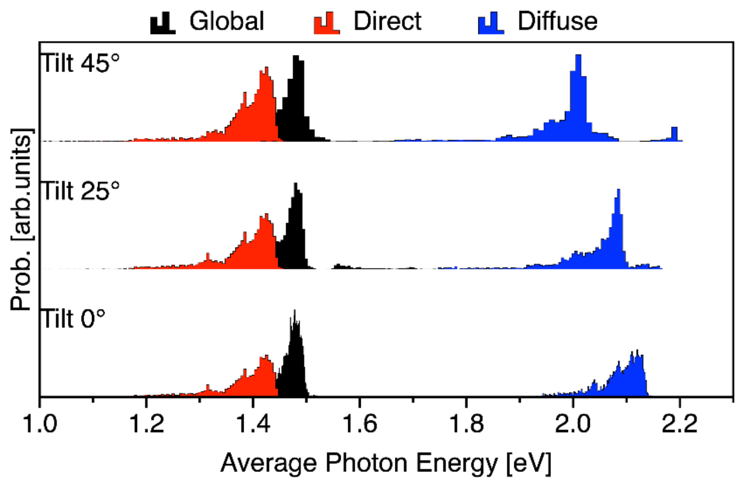

Figure S11. The average photon energy of global, direct, and diffuse solar radiation. The integration thresholds are 300nm and 4000nm[4].

The following figure reports the amount of diffuse light comparing different tilt angle. It can be observed that with a tilt of 0° there is a larger share of diffuse radiation in the winter months, and the opposite in summer. This combination of this aspect with the larger APE of the diffuse radiation explains the very low incidence of $\Delta J_{SC} < 0$ conditions with 0° tilt (see figure 5c).

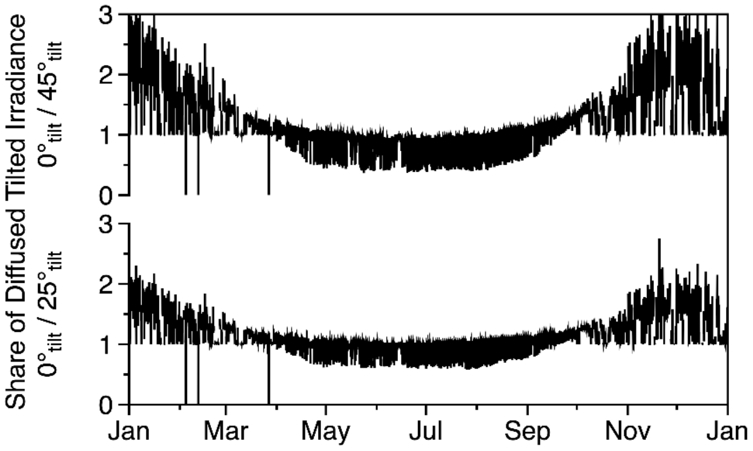

Figure S12. The relative amount of diffused radiation on the plane of a solar module. The comparison is between 0° and 25° or 45°. A value larger than 1 indicates that there is more diffuse light on the 0° plane in that specific hour.

For sake of clarity, the APE is evaluated by using the “clear sky” model provided by the pvlib python library and used to produce figure 3 of the main text. When comparing the “clear sky” model with the actual irradiance data provided by PVGIS, we maintained the clear sky spectral distribution. In this way we are overestimating the incidence of the $\Delta J_{SC} < 0$ conditions. In fact, in cloudy days the actual spectrum would be richer in the diffuse components, and thus richer in the short wavelength range, pushing the tandem solar cells towards $\Delta J_{SC} > 0$ conditions[5]. Here, we decided to maintain the same spectral shape of the “clear sky” model and simply scaling the total irradiance with the one from the typical meteorological year. In this way we place ourselves in the worst condition concerning the reverse bias protection from silicon, which would make sense to assess possible solution strategies. Overall, we do not expect a strong effect on the comparison between the different systems analyzed in this work.

Irradiance and Temperature Behavior.

The temperature of the solar cell has been calculated using the Ross model[6], through the equation:

$$T_{CELL} = \frac{NOCT - 20°C}{800W/m^2} G + T_{AIR}$$

We set a NOCT value of 44°C.

The temperature profile of the solar cell during the year is reported herein, for the case with tilt 25°C.

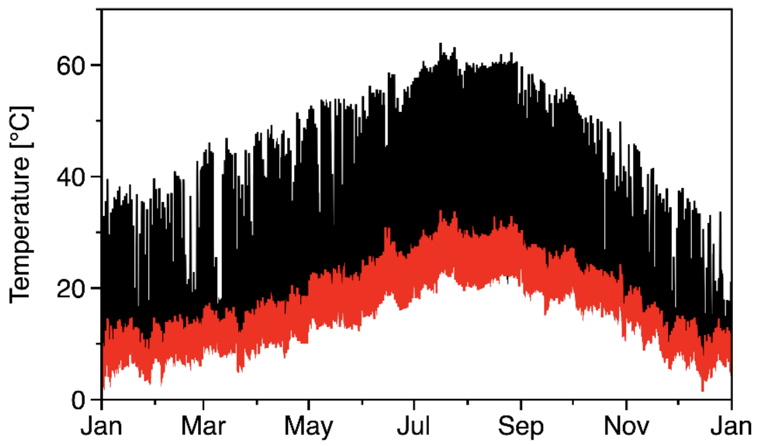

Figure S13. Air temperature and cell temperature across an entire year.

The temperature has been used to correct the Jsc of the two sub cells after the EQE integration with the solar spectra. For the perovskite we considered a Jsc thermal coefficient of -0.05 [rel-%/°C] and for silicon +0.05 [rel-%/°C] (the actual value for the silicon sub cell would be twice as large, benefiting also from the bandgap enlargement of the perovskite). [7–9] The temperature also affects the value of j0 for the two sub cells, evaluated as discussed in this reference.[10]

The impact of the temperature on Rs and Rsh is neglected. We do not expect a strong impact on this aspect in the comparison between the different system analysed in this work.

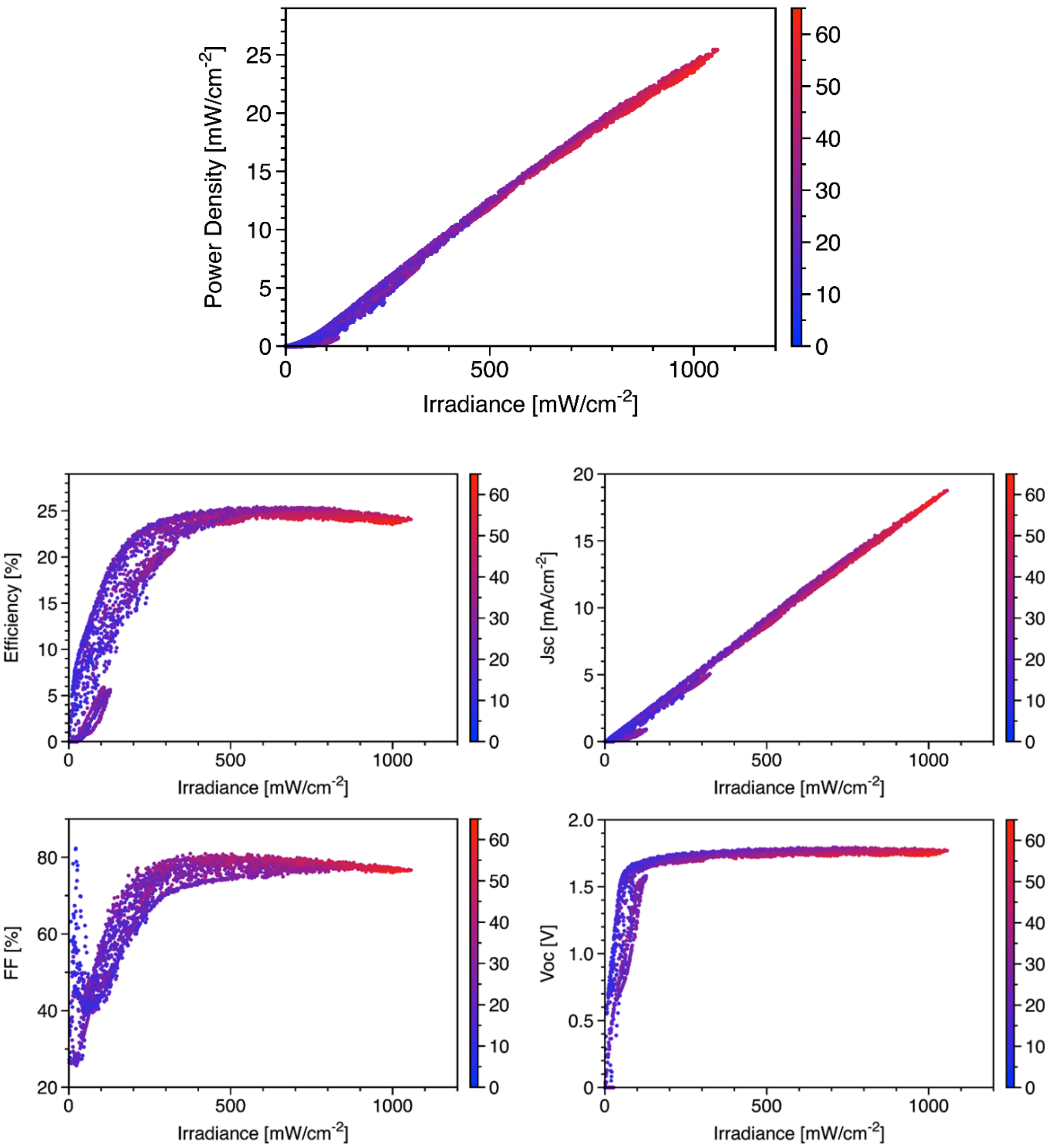

Figure S14. Irradiance dependance of power production and PV parameters. The color ramp refers to the temperature of the solar cell.

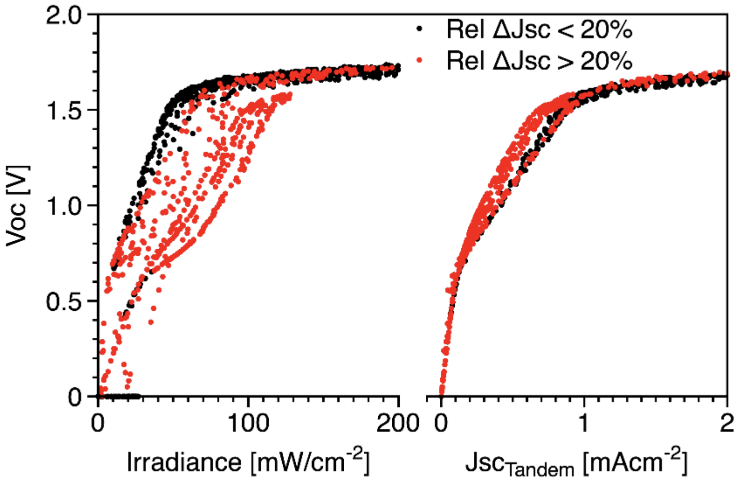

Figure S15. Low Irradiance Behavior. In the Voc distribution against the irradiance shown in figure S13, we noticed a large amount of points with a low Voc at relatively high irradiance values. Considering the logarithmic dependance of the Voc with the irradiance, this could be worthy a further analysis. In first place, this is not to be attributed to the temperature of the solar cell. In fact, there is a correlation between irradiance and temperature (also strengthen by the Ross model employed in our simulation). The explanation lies behind the effect of current mismatch at low irradiance level, which could bring the tandem cell in situation where one sub cell absorbs a not negligible amount of light, while the other sub cell remains in (pseudo) dark. A parameter describing this effect is the relative current mismatch, and we see that all the excessively low Voc values at about 100 mW/cm^2 of irradiance (about 0.1 Sun) are associated with a high relative current mismatch. On the right side of the figure, we plotted the Voc trend against the Jsc of the tandem (2mA/cm^2 roughly corresponds to 0.1Sun, see figure S13), which is a clear indicator of the minimum amount of light absorbed by either cell. In this figure, the behavior of the solar cell is more regular, confirming our explanation.

The effect discussed on the Voc, reflects on all the other PV parameters.

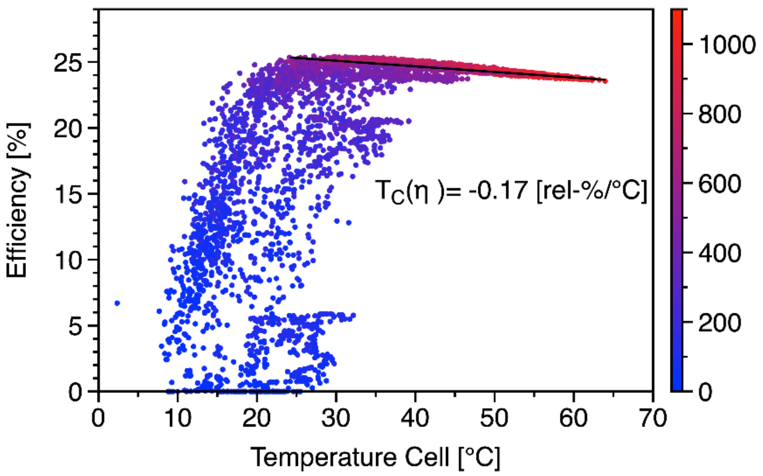

Figure S16. Temperature dependance of the power conversion efficiency of the solar cell. The color ramp refers to the irradiance (W/m^2). The thermal coefficient is obtained considering only values with an Irradiance above 500 W/m^2 (to neglect the effect of the drop of efficiency at low irradiance)

The temperature coefficient for the efficiency of the solar cell, considering the points with irradiance above 500 W/m^2 (where the trend of efficiency against irradiance is quite flat) is calculated as -0.17 relative % per °C. This value is in line with previous reports[1,11,12].

References

(1) Tomšič, Š.; Jošt, M.; Brecl, K.; Topič, M.; Lipovšek, B. Energy Yield Modeling for Optimization and Analysis of Perovskite-Silicon Tandem Solar Cells Under Realistic Outdoor Conditions. *Adv Theory Simul* **2023**, *6* (4). https://doi.org/10.1002/adts.202200931.

(2) Jošt, M.; Matič, G.; Köhnen, E.; Li, B.; Glažar, B.; Jankovec, M.; Albrecht, S.; Topič, M. Subcell Operation and Long-Term Stability Analysis of Perovskite-Based Tandem Solar Cells Using a Bichromatic Light Emitting Diode Light Source. *Solar RRL* **2021**, *5* (8). https://doi.org/10.1002/solr.202100311.

(3) Bowring, A. R.; Bertoluzzi, L.; O'Regan, B. C.; McGehee, M. D. Reverse Bias Behavior of Halide Perovskite Solar Cells. *Adv Energy Mater* **2018**, *8* (8). https://doi.org/10.1002/aenm.201702365.

(4) Schmager, R.; Langenhorst, M.; Lehr, J.; Lemmer, U.; Richards, B. S.; Paetzold, U. W. Methodology of Energy Yield Modelling of Perovskite-Based Multi-Junction Photovoltaics. *Opt Express* **2019**, *27* (8), A507. https://doi.org/10.1364/oe.27.00a507.

(5) Nann, S.; Riordan, C. Solar Spectral Irradiance under Clear and Cloudy Skies: Measurements and a Semiempirical Model. *Journal of Applied Meteorology* **1991**, *30* (4), 447–462. https://doi.org/10.1175/1520-0450(1991)030<0447:SSIUCA>2.0.CO;2.

(6) Ross, R. G. Jr. ,. Design Techniques for Flat-Plate Photovoltaic Arrays. In *15th IEEE Photovoltaic Specialist Conference*; Orlando, FL., 1981.

(7) Ponce-Alcantara, S.; Connolly, J. P.; Sanchez, G.; Miguez, J. M.; Hoffmann, V.; Ordas, R. A Statistical Analysis of the Temperature Coefficients of Industrial Silicon Solar Cells. In *Energy Procedia*; Elsevier Ltd, 2014; Vol. 55, pp 578–588. https://doi.org/10.1016/j.egypro.2014.08.029.

(8) Aydin, E.; Allen, T. G.; De Bastiani, M.; Xu, L.; Ávila, J.; Salvador, M.; Van Kerschaver, E.; De Wolf, S. Interplay between Temperature and Bandgap Energies on the Outdoor Performance of Perovskite/Silicon Tandem Solar Cells. *Nat Energy* **2020**, *5* (11), 851–859. https://doi.org/10.1038/s41560-020-00687-4.

(9) Dupré, O.; Vaillon, R.; Green, M. A. Physics of the Temperature Coefficients of Solar Cells. *Solar Energy Materials and Solar Cells* **2015**, *140*, 92–100. https://doi.org/10.1016/j.solmat.2015.03.025.

(10) Wang, S.; Wang, C.; Ge, Y.; Liu, S.; Xu, J.; Ahmed Amer, R. In-Depth Analysis of Photovoltaic Module Parameter Estimation. *Energy* **2024**, *291*. https://doi.org/10.1016/j.energy.2024.130345.

(11) Liu, H.; Rodríguez-Gallegos, C. D.; Liu, Z.; Buonassisi, T.; Reindl, T.; Peters, I. M. A Worldwide Theoretical Comparison of Outdoor Potential for Various Silicon-Based Tandem Module Architecture. *Cell Rep Phys Sci* **2020**, *1* (4). https://doi.org/10.1016/j.xcrp.2020.100037.

(12) Jošt, M.; Lipovšek, B.; Glažar, B.; Al-Ashouri, A.; Brecl, K.; Matič, G.; Magomedov, A.; Getautis, V.; Topič, M.; Albrecht, S. Perovskite Solar Cells Go Outdoors: Field Testing and Temperature Effects on Energy Yield. *Adv Energy Mater* **2020**, *10* (25). https://doi.org/10.1002/aenm.202000454.
